# Development and optimization of a new culture media using extruded bean as nitrogen source

**DOI:** 10.1016/j.mex.2015.03.001

**Published:** 2015-03-12

**Authors:** Karla A. Batista, Kátia F. Fernandes

**Affiliations:** Laboratório de Química de Polímeros, Departamento de Bioquímica e Biologia Molecular, Universidade Federal de Goiás, Goiânia, GO, Brazil

**Keywords:** Extruded bean, Mixture design, *S. cerevisiae*, *P. pastoris*, Mathematical modeling

## Abstract

The composition of a culture medium is one of the most important parameters to be analyzed in biotechnological processes with industrial purposes, because around 30–40% of the production costs were estimated to be accounted for the cost of the growth medium [1]. Since medium optimization using a one-factor-at-a-time approach is time-consuming, expensive, and often leads to misinterpretation of results, statistical experimental design has been applied to medium optimization for growth and metabolite production [2], [3], [4], [5]. In this scenario, the use of mixture design to develop a culture medium containing a cheaper nitrogen source seems to be more appropriate and simple. In this sense, the focus of this work is to present a detailed description of the steps involved in the development of a optimized culture medium containing extruded bean as nitrogen source.

• In a previous work we tested a development of new culture media based on the composition of YPD medium, aiming to reduce bioprocess costs as well as to improve the biomass production and heterologous expression.
• The developed medium was tested for growth of *Saccharomyces cerevisiae* and *Pichia pastoris* (GS 115).
• The use of culture media containing extruded bean as sole nitrogen source showed better biomass production and protein expression than those observed in the standard YPD medium.

## Graphical abstract

**Figure:**
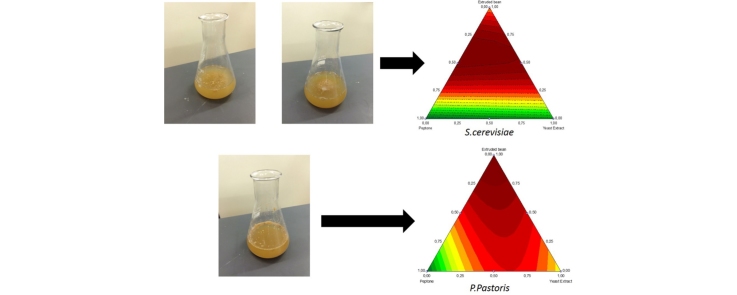

## Method details

Briefly, the development of the new culture media containing extruded bean as substitute of traditional nitrogen source (peptone and yeast extract) involved the following steps:

(1) Production of the extruded bean flour.
(2) Use of statistical approach to optimize the composition of nitrogen source.
(3) Evaluation of the yeast growth profile in the new media compared to a commercial YPD media.
(4) Estimation of the potential use of the new culture media for heterologous expression.

### Production of extruded bean flour

Hard-to-cook common beans (*Phaseolus vulgaris*, cv carioca) were ground in a hammer mill and sifted in a screen of 0.425 mm. Then, the moisture of the bean flour was adjusted to 25% and the flour was kept out at refrigerator (4 °C) overnight aiming to reach a wetting balance. Before extrusion, the bean flour was kept out at room temperature for 2 h and the moisture content was confirmed gravimetrically [6]. The extrusion process was carried out using a Cerealtec International single screw extruder (CT-L15), with four zones of heating, compression ratio screw of 3:1, 5 mm die and screw speed of 150 rpm. The temperatures used in each heating zone of the extruder were 80 °C, 140 °C, 150 °C and 130 °C [7], [8]. The extrudates were dried at room temperature for 12 h, milled and sifted in a screen of 0.425 mm. This extruded bean flour was used as nitrogen source in the culture media.

### Mathematical model determination for optimization of culture media

The initial composition of culture media was based on the commercial medium YPD, widely used for yeasts growth. This medium is composed by 1% yeast extract, 2% peptone and 2% dextrose. So, in order to evaluate the effectiveness of substituting peptone and yeast extract for extruded bean, the composition of the culture media was optimized using a 3-factor simplex-lattice design. This mixture design was used to study the relationship between the proportion of the different nitrogen sources and their respective responses in the optical density of *Saccharomyces cerevisiae* and *Pichia pastoris* (GS 115). The design was implemented using Statistica 7.0 software (StatSoft Inc., Tulsa, OK, USA) and the proportion of each nitrogen source varied from 0 to 2% (Table 1).

### Media preparation

The preparation of culture media containing extruded bean as nitrogen source was carried out considering the percentage of substitution determined by the mathematical design. Considering that extruded bean is a complex material not completely soluble, after dissolution of all components and before autoclaving, the medium was filtered through a cellulosic membrane (gauze) to avoid the presence of fibrous insoluble materials.

### Effectiveness of culture media for growth of yeasts

The different culture media proposed by mixture design were tested for the growth of *S. cerevisiae* and *P. pastoris* (GS 115). Prior to tests pre-inoculums were done as follows: 5 mL of YPD medium was prepared and 20 μL of a culture containing 2 × 10^4^ cells was inoculated. The pre-inoculum of *S. cerevisiae* was incubated at 30 °C for 24 h under shaking at 150 rpm. Regarding to *P. pastoris* (GS 115), the pre-inoculum was incubated at 37 °C for 48 h under shaking at 150 rpm. After incubation, cells were centrifuged at 4 °C for 10 min at 5000 g and the pellet was washed with 1 mL of sterile saline solution (0.15 mol L^−1^). Then, the absorbance of cell suspension was adjusted to 0.500 (*λ* 600 nm), using sterile saline solution (0.15 mol L^−1^) and 250 μL of cell suspension of *S. cerevisiae* or *P. pastoris* (GS115) strains were transferred to flasks containing 250 mL of the different media.

**Fig. 1:**
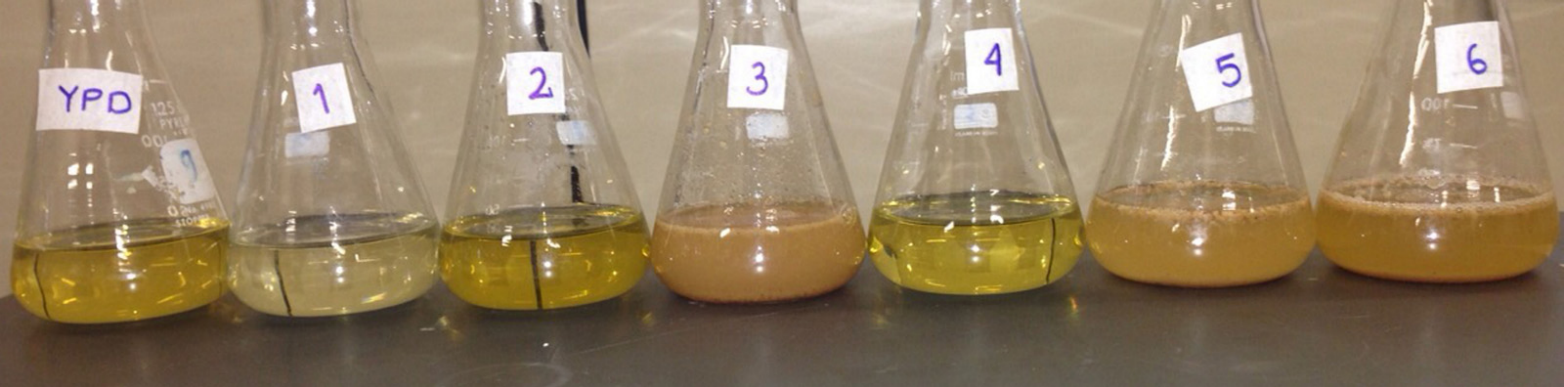
Overview of the different culture media developed using mixture design approach.

Aiming to evaluate the growth profile of *S. cerevisiae,* the culture media were incubated at 37 °C and 150 rpm and aliquots of 1 mL were withdraw every 2 h until the stabilization of the optical density at 600 nm. For *P. pastoris* (GS 115), which presents a slower growth rate, the culture media were incubated at 30 °C and 150 rpm and the aliquots were withdraw every 4 h. Aliquots were centrifuged at 4 °C for 10 min at 5000 × *g* and the cell mass was re-suspended in 1 mL sterile saline solution (0.15 mol L^−1^). The optical density of cell suspension was determined at 600 nm by using a UV–vis spectrophotometer. Considering that culture media containing extruded bean are colored mixtures (Fig. 1), blanks were made with respective culture media without inoculum.

The results of growth evidenced that the best medium composition for growth of *S. cerevisiae* was that containing 1% extruded bean flour and 1% peptone (experiment 5) or 1% extruded bean flour and 1% yeast extract (experiment 6) plus 2% dextrose. In the other hand, for *P. pastoris* (GS 115), the best composition was found as 2% of extruded bean flour (experiment 3) plus 2% dextrose.

### Protein expression using *P. pastoris* (GS 115)

Considering that high values of growth profile do not necessarily mean a proportional protein expression, the effectiveness of heterologous expression by *P. pastoris* (GS 115) was tested in medium containing 2% of extruded bean flour. The setup of tests for recombinant protein expression using *P. pastoris* (GS 115) was carried out as recommended by the supplier (Invitrogen, Carlsbad, CA). Starter cultures were prepared through inoculation of 1 mL of *P. pastoris* (GS 115) culture (10^6^ cells) in 100 mL of YPD medium, incubated at 30 °C and 150 rpm for 24 h. After this initial growth phase, 20 mL of the recombinant culture was harvested by centrifugation (10,000 × *g*, 5 min) and cell pellet was washed twice with 10 mL of 100 mmol L^−1^ sodium phosphate buffer (pH 6.0). The washed recombinant *P. pastoris* (GS 115) cells were re-suspended in 20 mL of the expression medium supplemented with 1 mL of 20% (v/v) methanol, prepared with sterile milliQ water.

The protein expression by *P. pastoris* (GS 115) was tested using the standard medium (1% yeast extract, 2% peptone, 2% dextrose and 3% glycerol (UtraPure™, Invitrogen)), and the medium containing extruded bean (2% extruded bean, 2% dextrose and 3% glycerol). The expression systems were incubated at 30 °C for 96 h at 150 rpm, with the addition of 1 mL of 20% methanol (prepared with sterile milliQ water) every 24 h.

Aliquots of 1 mL of culture were collected every 24 h and analyzed for the production of extracellular protein. The cell-free supernatant from the expression medium was recovered by centrifugation at 4 °C for 10 min at 5000 × *g*. Total proteins were subsequently precipitated with cold acetone (1:2 v/v), incubated at −80 °C for 2 h, centrifuged for 5 min (4 °C and 5000 × *g*) and re-suspended in 100 μL of 100 mmol L^−1^ sodium phosphate buffer (pH 6.0). Total protein concentration was determined following the method described by Bradford [9].

The precipitated proteins were analyzed for purity by using 10% denaturizing polyacrylamide gel electrophoresis (SDS-PAGE) [10]. The protein samples were prepared by mixing 2× (double strength) gel loading buffer (10 mM Tris-HCl pH 6.8, 4% SDS, 0.2% bromophenol blue, 20% glycerol) and 10% β-mercaptoethanol mixed in the proportion of 1:1, boiled for 5 min, centrifuged (10,000 × *g*) for 2 min, and then loaded onto gel. The total protein was calculated to be approximately 20 μg. Electrophoresis was run at 25–40 mA for 2 h at room temperature. Gels were stained with Comassie Blue R-250. Pre-stained molecular weight markers (New England Biolabs, MA) ranging from 7 to 175 kDa were used as a running standard. The image analysis of the gel was performed by using the program ImageJ 1.46r (Wayne Rasband, National Institute of Health, USA). The parameters for obtaining/recording the gel image were 300 dpi-resolution, 100%-zoom, and 16 bits per pixel-depth.

## Statistical analysis

The statistical analysis of the experimental mixture design was performed by multivariate analysis. The model was simplified to exclude terms that were not considered statistically significant (*p* > 0.05) by analysis of variance (ANOVA). The quality of the polynomial model equation was judged by using the coefficient of determination *r*^2^.

The response function (*Y*) of the mixture model was explained by the following equation:

(1) $$y=\overset{q}{\underset{i=1}{\sum }}\beta_{i}x_{i}+\sum_{}^{}\sum_{i<j}^{q}\beta_{ij}x_{i}x_{j}$$

Geometrically, in Eq. (1) the parameter *β_i_* represents the expected response of the pure mixture *x_i_* = 1, *x_j_* = 0, *j* ≠ *i*. The term given by $y=\sum_{i=1}^{q}\beta_{i}x_{i}$ represents the response when blending is strictly additives and there are no interactions between the components of the mixture. The quadratic term *β_ij_x_i_x_j_* represents the excess response over the linear model due to interaction between two components [11].

The mixture design and all subsequent tests were conducted in triplicate and the level of significance was 95%. All analyses were carried out by using the software Statistica 7.0 (StatSoft Inc., Tulsa, OK, USA).

## Figures and Tables

**Table 1:** Mixture design and assigned concentrations of each compound at different levels of the mixture design.

Experiment	Peptone (X1)	Yeast extract (X2)	Extruded bean (X3)
1	2%	0	0
2	0	2%	0
3	0	0	2
4	1%	1%	0
5	1%	0	1%
6	0	1%	1%
